# Author Correction: Single-nuclei and bulk-tissue gene-expression analysis of pheochromocytoma and paraganglioma links disease subtypes with tumor microenvironment

**DOI:** 10.1038/s41467-022-35751-y

**Published:** 2023-01-09

**Authors:** Magnus Zethoven, Luciano Martelotto, Andrew Pattison, Blake Bowen, Shiva Balachander, Aidan Flynn, Fernando J. Rossello, Annette Hogg, Julie A. Miller, Zdenek Frysak, Sean Grimmond, Lauren Fishbein, Arthur S. Tischler, Anthony J. Gill, Rodney J. Hicks, Patricia L. M. Dahia, Roderick Clifton-Bligh, Karel Pacak, Richard W. Tothill

**Affiliations:** 1 Peter MacCallum Cancer Centre, Melbourne, VIC Australia; 2 Centre for Cancer Research and Department of Clinical Pathology, University of Melbourne, Melbourne, VIC Australia; 3 Department of Surgery, Royal Melbourne Hospital, Parkville, VIC Australia; 4 Department of Surgery, Epworth Hospital, Richmond, VIC Australia; 5 3rd Department of Internal Medicine—Nephrology, Rheumatology and Endocrinology, Faculty of Medicine and Dentistry, Palacky University Olomouc and University Hospital Olomouc, Olomouc, Czech Republic; 6 Department of Medicine, Division of Endocrinology, Metabolism, Diabetes, University of Colorado, Aurora, CO USA; 7 Tufts Medical Centre, Boston, MA USA; 8 Sydney Medical School, University of Sydney, Sydney, NSW Australia; 9 Kolling Institute of Medical Research, Royal North Shore Hospital, Sydney, NSW Australia; 10 NSW Health Pathology, Department of Anatomical Pathology, Royal North Shore Hospital, Sydney, NSW Australia; 11 Div. Hematology and Medical Oncology, Department of Medicine, Mays Cancer Center, University of Texas Health Science Center at San Antonio (UTHSCSA), San Antonio, TX USA; 12 Eunice Kennedy Shriver National Institute of Child Health and Human Development, Bethesda, MD USA; 13 Sir Peter MacCallum Department of Oncology, University of Melbourne, Melbourne, VIC Australia

**Keywords:** Neuroendocrine cancer, Cancer genomics, Adrenal tumours

Correction to: *Nature Communications* 10.1038/s41467-022-34011-3, published online 21 October 2022

The original version of this Article contained an error in Supplementary Data 5, as column A listing the gene names was misaligned with the information presented in the other columns (B-H). The HTML has been updated to include the corrected version of Supplementary Data 5.

## Supplementary information

Updated Supplementary Data 5

