# Supplementary material for: Single-nuclei and bulk-tissue gene-expression analysis of pheochromocytoma and paraganglioma links disease subtypes with tumor microenvironment
Source: Nat Commun. 2022 Oct 21;13:6262. doi: 10.1038/s41467-022-34011-3 (PMC9587261; doi:10.1038/s41467-022-34011-3)
Supplement: Supplementary file 1 — Supplementary Information [file 41467_2022_34011_MOESM1_ESM.pdf]

Supplementary Table 1. Association between PCPG subtype and metastasis (Fisher's exact test, Benjamini-Hochberg multiple testing correction)

Subtype	n_metastatic	n_non-metastatic	odds_ratio	p.value	p.adjust
C1A1 (SDHx)	23	31	8.5091868	7.77E-10	6.22E-09
C1A2 (SDHx-HN)	8	9	7.22947326	3.18E-04	8.47E-04
C1B1 (VHL)	8	69	0.79654021	0.70287852	0.70287852
C1B2 (EPAS1)	0	19	0	0.148234332	0.169410665
C2A (Kinase)	7	145	0.24517765	1.94E-04	7.76E-04
C2B1 (MAX)	2	48	0.27015295	0.065708259	0.087611012
C2B2 (MAML3)	4	9	3.33292785	0.062663338	0.087611012
C2C	1	48	0.13254447	0.018560746	0.037121492

Supplementary Table 2. IHC scoring in 12 PCPG and 2 normal adrenal tissues

SampleID	ID Number	Subtype	Genotype	CD3 per $2mm^2$	CD68 per $2mm^2$	CD163 per $2mm^2$	CD206 per $2mm^2$
E041	VPH36T	C1A1 (SDHx)	SDHA	3	9	15	4
E019	VPH23T	C1A1 (SDHx)	SDHB	6	18	40	12
P018-PGL1	P018P1.2.2	C1A1 (SDHx)	SDHB	5	42	50	9
P018-PGL3	P018M3	C1A1 (SDHx)	SDHB	1	12	70	55
E024	VPH35T	C1B1 (VHL)	VHL	3	4	150	150
E041	07SH0498	C1B1 (VHL)	VHL	5	60	250	240
E042	18SH0221	C1B1 (VHL)	VHL	15	24	185	175
E035	07AH0391	C2A (Kinase)	HRAS	3	44	150	125
E017	VPH20T	C2A (Kinase)	NF1	10	45	50	25
E174	17NH0056T1	C2A (Kinase)	NF1	1	2	25	12
E007	VPH08T	C2A (Kinase)	TMEM127	1	2	1	4
E008	VPH09T	C2B2 (MAML3)	UBTF-MAML3	5	3	120	25
Normal 1	Adrenal 22P5165	NAM	Normal	12	35	75	25
Normal 2	Adrenal	NAM	Normal	4	30	150	95

Supplementary Table 3. Differentially expressed genes in metastatic PCPG compared to non-metastatic PCPG. Differential gene expression was determined by limma using empirical Bayes moderated t-statistics, and adjusted for FDR with Benjamini-Hochberg correction (BH adj. P-value < 0.05)

Gene	logFC	AveExpr	t	P.Value	adj.P.Val	B	Pathways Groups	Comparison
JANSKY_cycling_neuroblast_genes	0.6338	0.0156	4.82	2.10E-06	0.0001	4.5086	Proliferation	metastatic_vs_nonmetastatic_in_C1A
HALLMARK_G2M_CHECKPOINT	0.4212	-0.0214	4.2722	2.46E-05	0.0008	2.1926	Proliferation	metastatic_vs_nonmetastatic_in_C1A
ZETHOVEN_SCLCs	-0.442	0.0037	-3.6199	0.0003	0.0075	-0.233	SCLCs	metastatic_vs_nonmetastatic_in_C1A
HALLMARK_MITOTIC_SPINDLE	0.1859	-0.057	3.0867	0.0022	0.0342	-1.9378	Proliferation	metastatic_vs_nonmetastatic_in_C1A
HALLMARK_E2F_TARGETS	0.2718	-0.0776	3.008	0.0028	0.0342	-2.1677	Proliferation	metastatic_vs_nonmetastatic_in_C1A
JANSKY_late_SCP_genes	-0.3055	0.0025	-2.9814	0.0031	0.0342	-2.2441	SCLCs	metastatic_vs_nonmetastatic_in_C1A
HALLMARK_GLYCOLYSIS	0.1594	-0.0536	2.7989	0.0054	0.0498	-2.7508	Other	metastatic_vs_nonmetastatic_in_C1A
JANSKY_early_SCP_genes	-0.2596	0.0043	-2.7665	0.006	0.0498	-2.8377	SCLCs	metastatic_vs_nonmetastatic_in_C1A

Supplementary Table 4. IHC antibodies and antigen retrieval methods

Antibody	Species	Distributor	Catalogue#	Dilution	Secondary	Antigen retrieval
CD3 (SP7)	Rabbit	Abcam	Ab16669	1:200	anti-Rabbit (ImmPRESS Vector labs)	Citrate Buffer, pH6
CD68 (514H12)	Mouse	Thermofisher Scientific	MA1-80133	1:100	anti-Mouse (ImmPRESS Vector labs)	Tris EDTA, pH9
CD206	Rabbit	Abcam	Ab64693	1:10000	anti-Rabbit(ImmPRESS Vector labs)	Citrate Buffer, pH6
CD163 (MRQ-26)	Mouse	Millipore Sigma	CM163M14	1:200	anti-Mouse (ImmPRESS Vector labs)	Tris EDTA, pH9
S100	Rabbit	Dako	Z3011	1:1000	anti-Rabbit (ImmPRESS Vector labs)	Tris EDTA, pH9

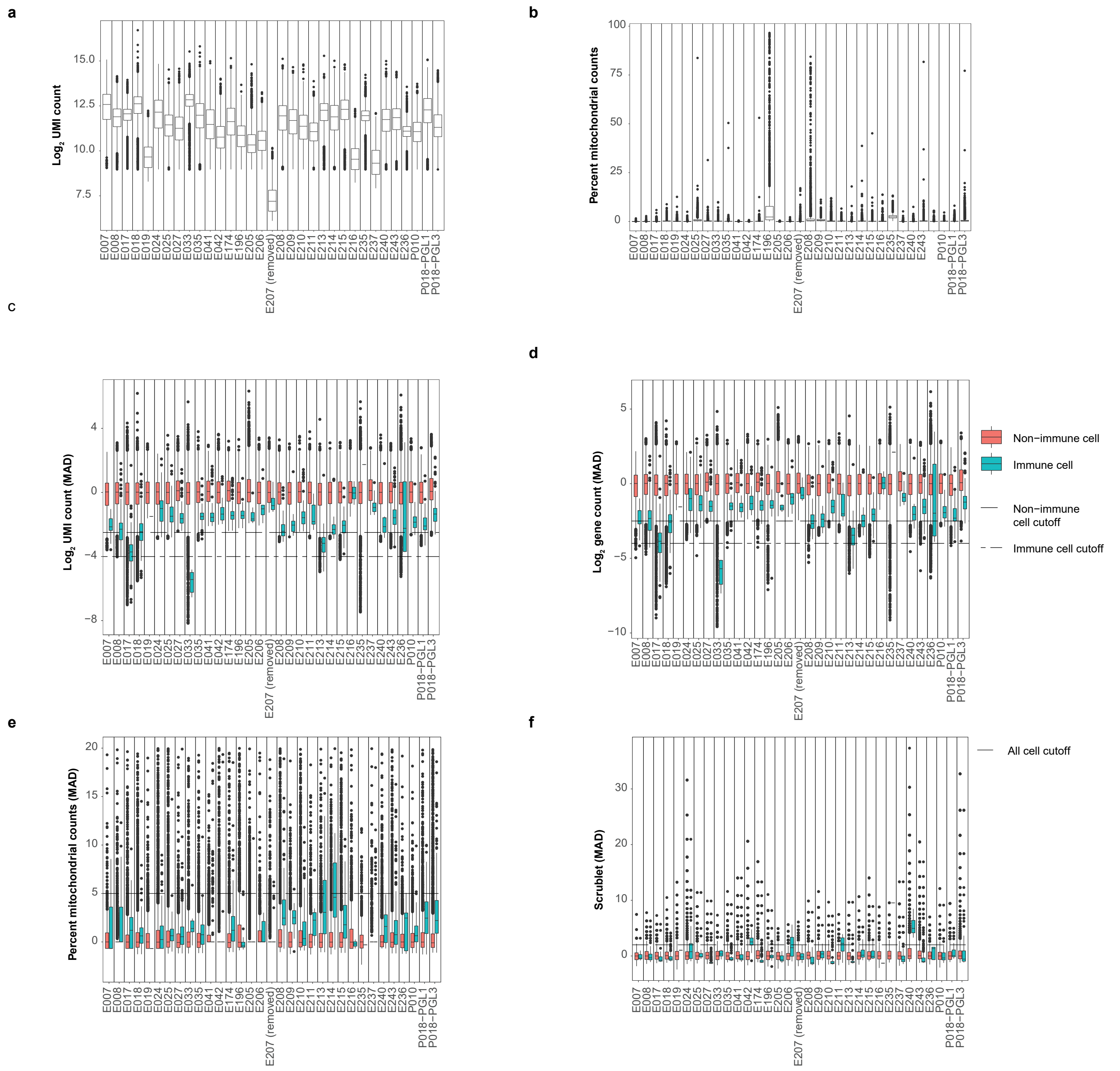

Supplementary Figure 1. QC and QC cutoffs of snRNA-Seq samples. **a**, Log$_2$ UMI counts of each sequenced sample (E207 was removed due to low library complexity). **b**, Per-nuclei percent mitochondrial read counts for each sample. Some samples displayed high global mitochondrial counts so an absolute filter threshold was not used. **c**, Median absolute deviation (MAD) Log$_2$ UMI counts for each sample, separated into non-monocyte-derived immune cell categories (based on scMatch) and otherwise per sample. A threshold of -4 was used for the immune cell group and -2.5 otherwise. **d**, MAD Log$_2$ gene counts for the same groups as (**c**). The same filter thresholds were used. **e**, MAD percent mitochondrial count values of each sample. Cells above 5 MADs were filtered. **f**, MAD Scrublet values. A threshold of 2 was chosen based on inspection of clusters showing intermediate gene expression and high average Scrublet scores. **a-f**, The lower and upper hinges of each boxplot correspond to the first and third quartiles, respectively, and the median value is marked. The whiskers extend from the hinges to the largest and smallest value no greater than 1.5 times the interquartile range above or below the upper and lower hinges, respectively. Values beyond the whisker extents are deemed outliers and are plotted individually. Number of samples=33; Number of cells per sample: E007=1040, E008=5214, E017=5540, E018=5177, E019=4534, E024=4902, E025=4295, E027=3943, E033=4162, E035=5190, E041=3493, E042=3154, E174=3987, E196=2856, E205=4111, E206=2962, E207=3432, E208=4105, E209=3374, E210=2952, E211=4435, E213=3617, E214=3706, E215=3787, E216=3230, E235=4140, E237=1596, E240=5519, E243=4672, E326=4717, P010=3653, P018-PGL1=4715, P018-PGL3=4729.

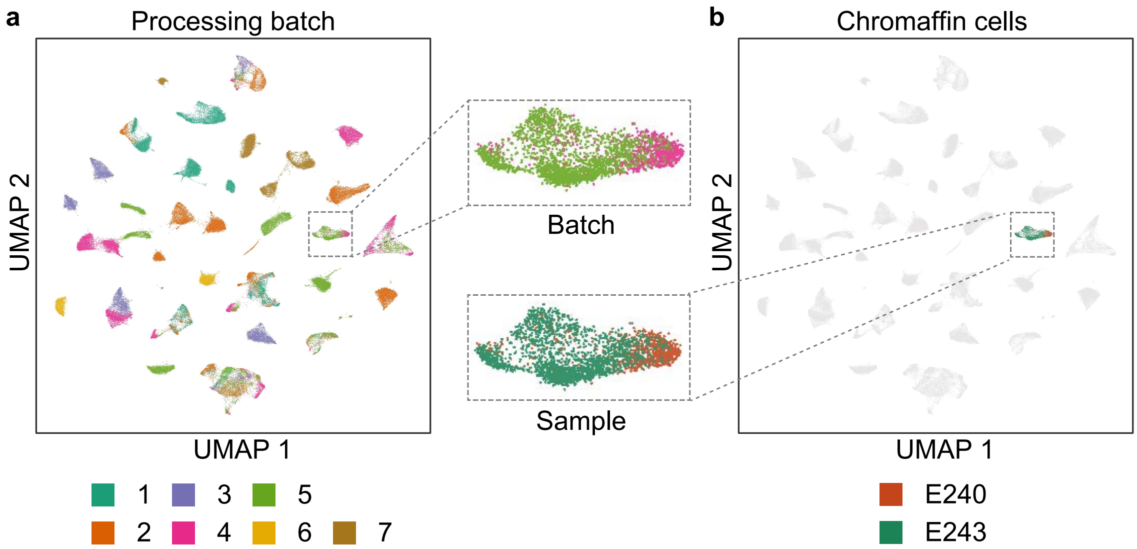

Supplementary Figure 2. UMAP projections for uncorrected PCPG snRNA-seq data show patient-specific clustering of PCPG tumor cells but not normal cells. **a**, UMAP colored by processing batch. **b**, The same UMAP as (a), colored by sample of origin, with cell types other than normal chromaffin cells colored grey.

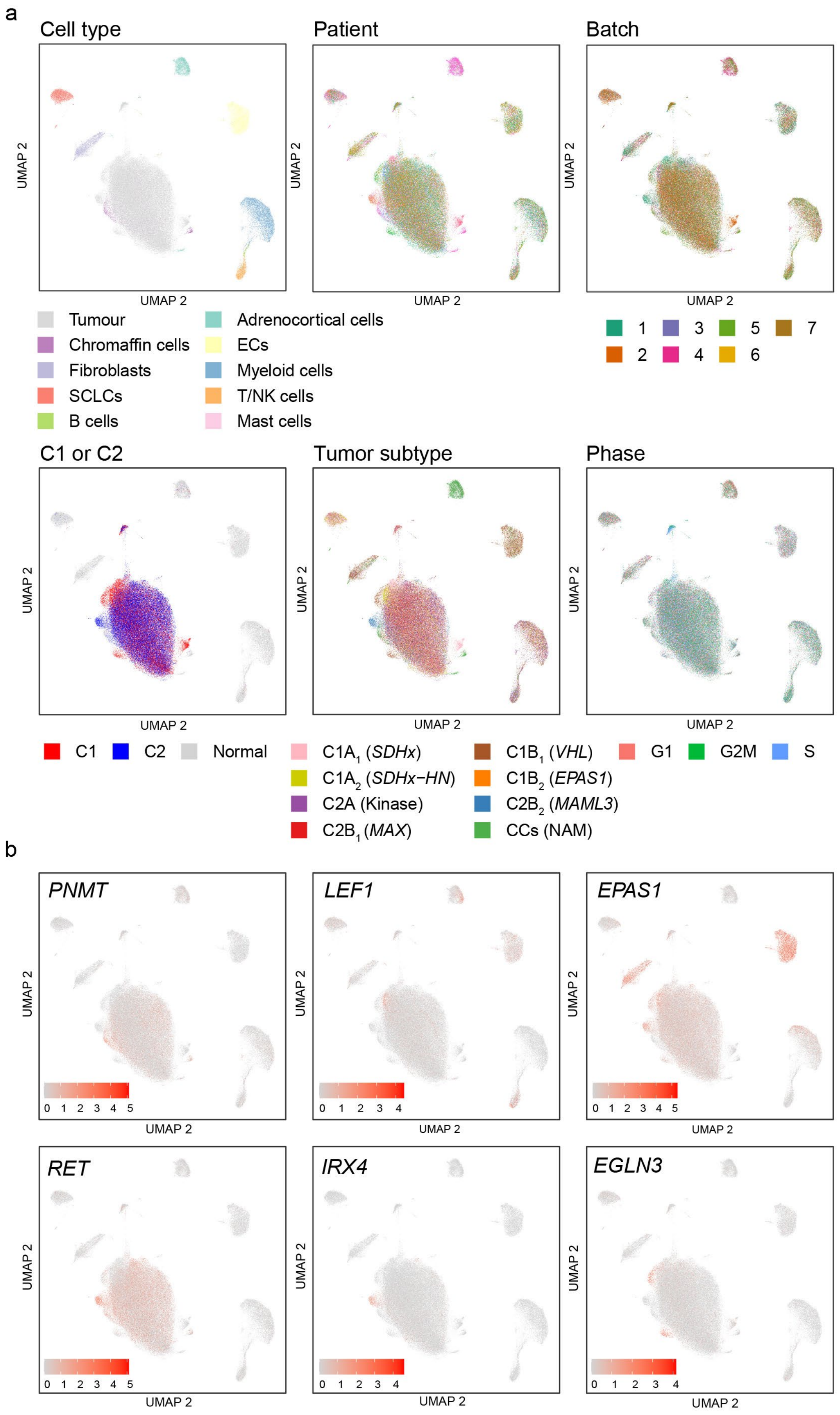

Supplementary Figure 3. Using Harmony batch correction to remove patient-specific effects in PCPG snRNA-seq. **a**, UMAP projections for batch-corrected PCPG snRNA-seq data, colored by different metadata as indicated by the plot subtitles. **b**, Feature plots of gene expression of cell type and tumor subtype marker genes.

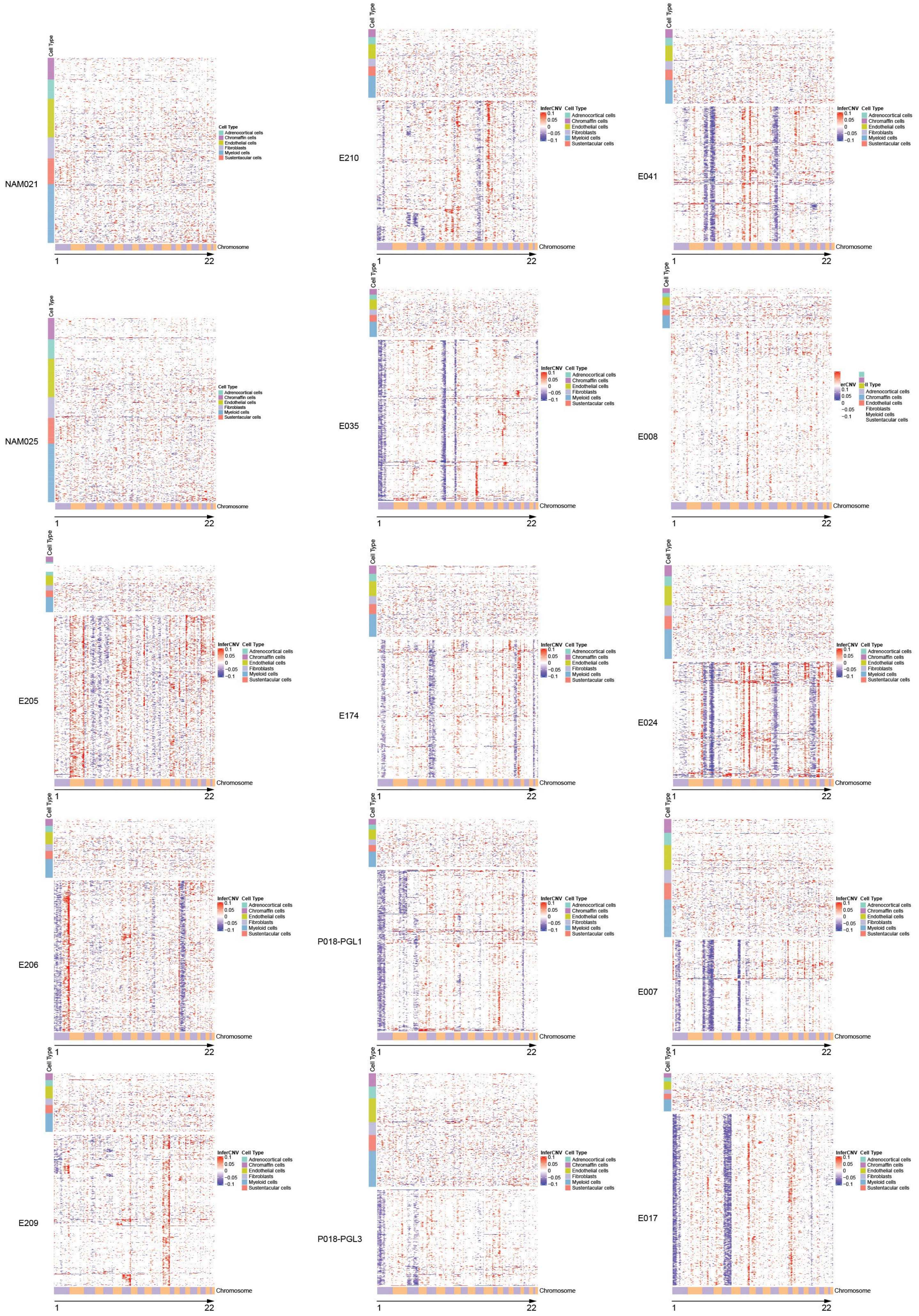
NAM021
E210
E041
NAM025
E035
E008
E205
E174
E024
E206
P018-PGL1
E007
E209
P018-PGL3
E017
Cell Type
InferCNV
0.1
0.05
0
−0.05
−0.1
Adrenocortical cells
Chromaffin cells
Endothelial cells
Fibroblasts
Myeloid cells
Sustentacular cells
Chromosome
1
22

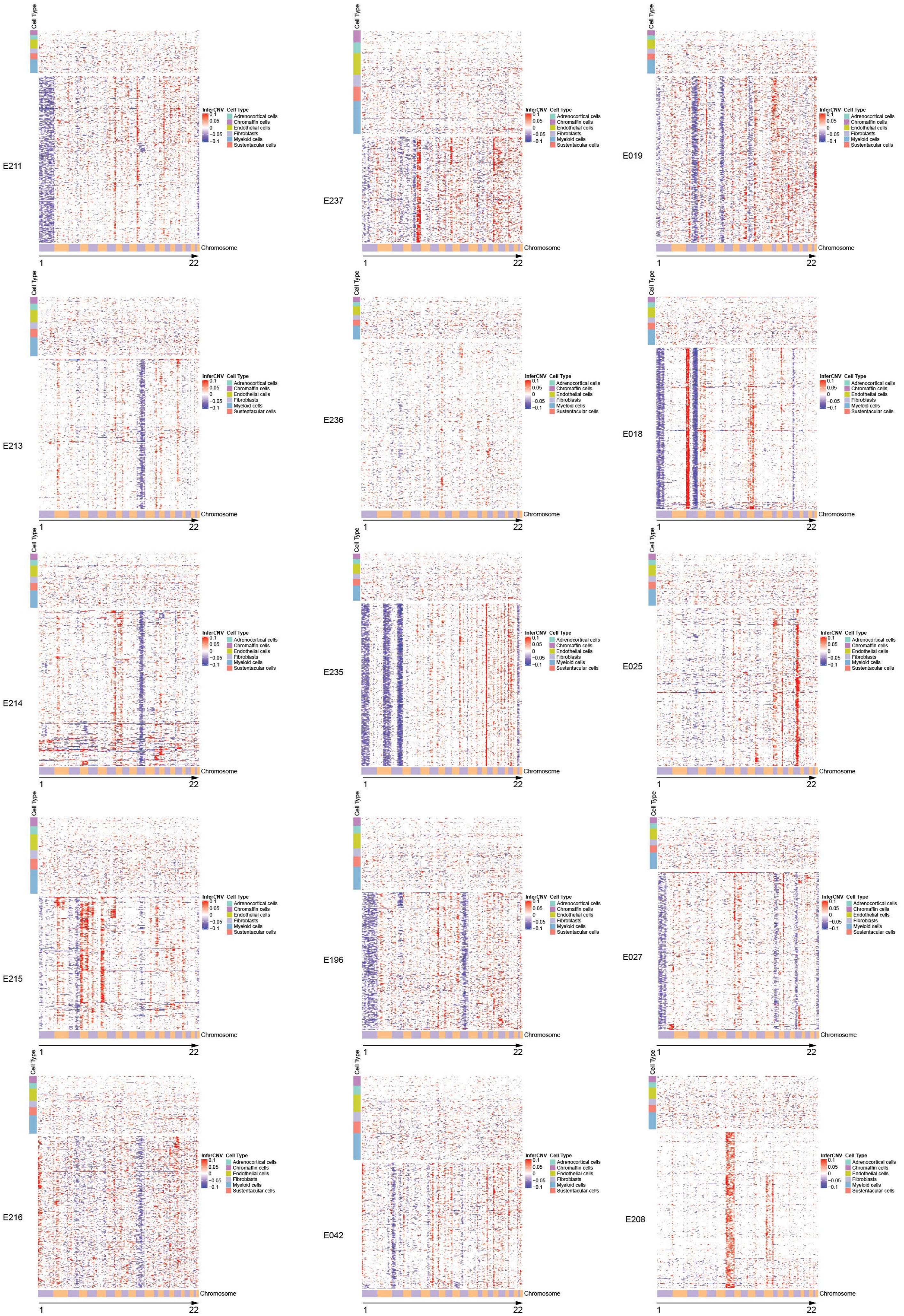
E211
E237
E019
E213
E236
E018
E214
E235
E025
E215
E196
E027
E216
E042
E208
Cell Type
InferCNV
0.1
0.05
0
−0.05
−0.1
Adrenocortical cells
Chromaffin cells
Endothelial cells
Fibroblasts
Myeloid cells
Sustentacular cells
Chromosome
1
22

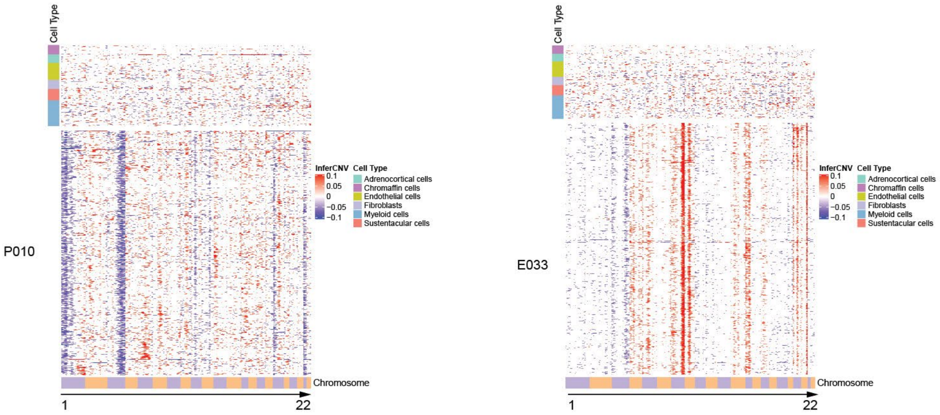

Supplementary Figure 4. Inference of copy-number in PCPG and normal adrenal snRNA-seq. Both NEO and non-NEO cell types are shown (bottom panel; tumor cells, top panel; non-neoplastic cell types from all tumors and NAM tissues).

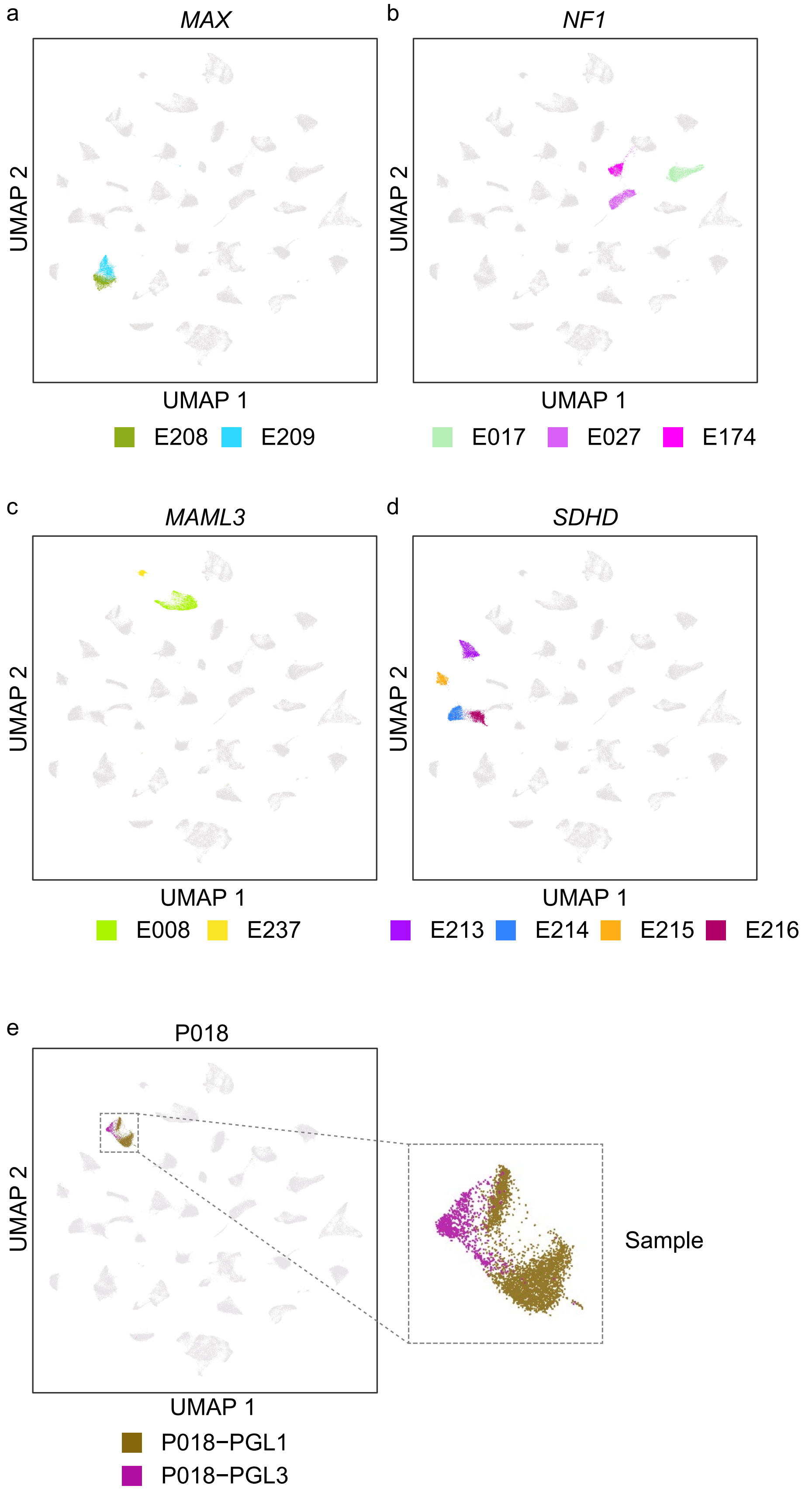

Supplementary Figure 5. UMAP projections for uncorrected PCPG snRNA-seq data show co-clustering of PCPG tumor samples based on PCPG driver mutations and tight synchronous primary PCPG tumors. **a-d**, UMAP projections each highlighting clustering of tumor nuclei for a specific tumor genotype. Nuclei from a single tumor genotype are colored according to sample of origin. Non-tumor nuclei and nuclei not belonging to the specified genotype are colored grey. **e**, UMAP highlighting tight co-clustering of synchronous primary PCPG samples collected from the same patient, tumor cells from patient P018 are colored according to tumor sample and cells from patients other than P018 are coloured grey.

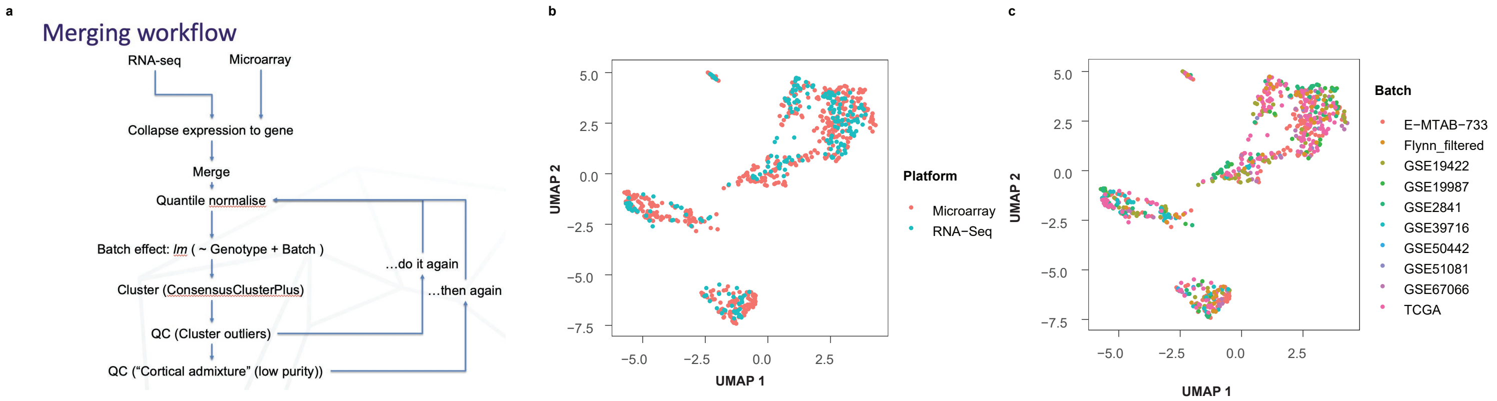

Supplementary Figure 6. Creation of the bulk RNA compendium **a**, Schematic description of data pooling and harmonization. Initial clustering attempts identified a cluster that was associated with datasets GSE19987 and GSE2841 but not of any particular genotypes. The cluster did not have a clear gene signature and had significantly higher than average normalized unscaled standard errors (NUSE), so these samples were deemed to be low-quality arrays and removed from the analysis before the merging process was repeated. A final consensus cluster number of nine was chosen based on the point at which the proportion of ambiguously clustered pairs stopped changing significantly. Additionally, a cluster associated with a normal cell gene signature and not any particular genotypes (C2C) was identified. Since this was a confounding factor in the batch effect removal model, the batch effect removal process was repeated a third time with samples initially assigned to cluster C2C to zero weight to improve performance. Two clusters associated with the kinase genotypes (C2A) were merged, yielding the final eight PCPG clusters. UMAPs with samples coloured by platform

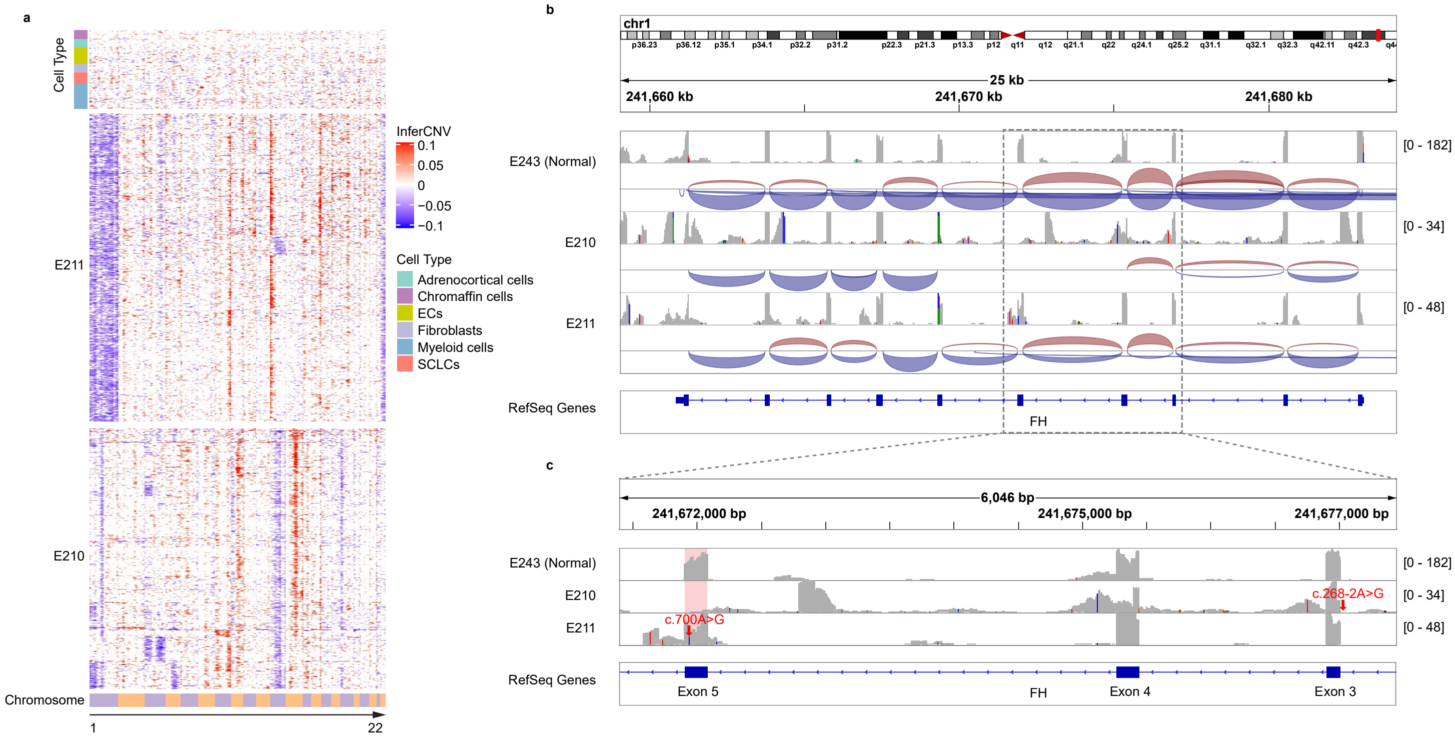

Supplementary Figure 7. snRNA-seq evidence for *FH* mutations in PCPG. a. Inferred CNV heatmaps for NEO cells for the *FH*-mutant samples (E210, E211) and normal cell types. b,c. Genome browser view of snRNA-seq read pileups for *FH* mutant samples and normal adrenal medulla control (E243). Reads from all cells (including NEO and normal cell types) are shown. b. snRNA-seq coverage for the entire *FH* gene, with sashimi plot illustrating splice junction. c. Zoomed-in coverage of mutations in *FH*. A c.268-2A>G mutation had previously been identified in E210 and this sample had evidence of disrupted splicing with reduced coverage at exon 5 (region highlighted in red). snRNA-seq reads confirmed presence of a c.700A>G mutation in E211. *FH* mutation positions are shown by

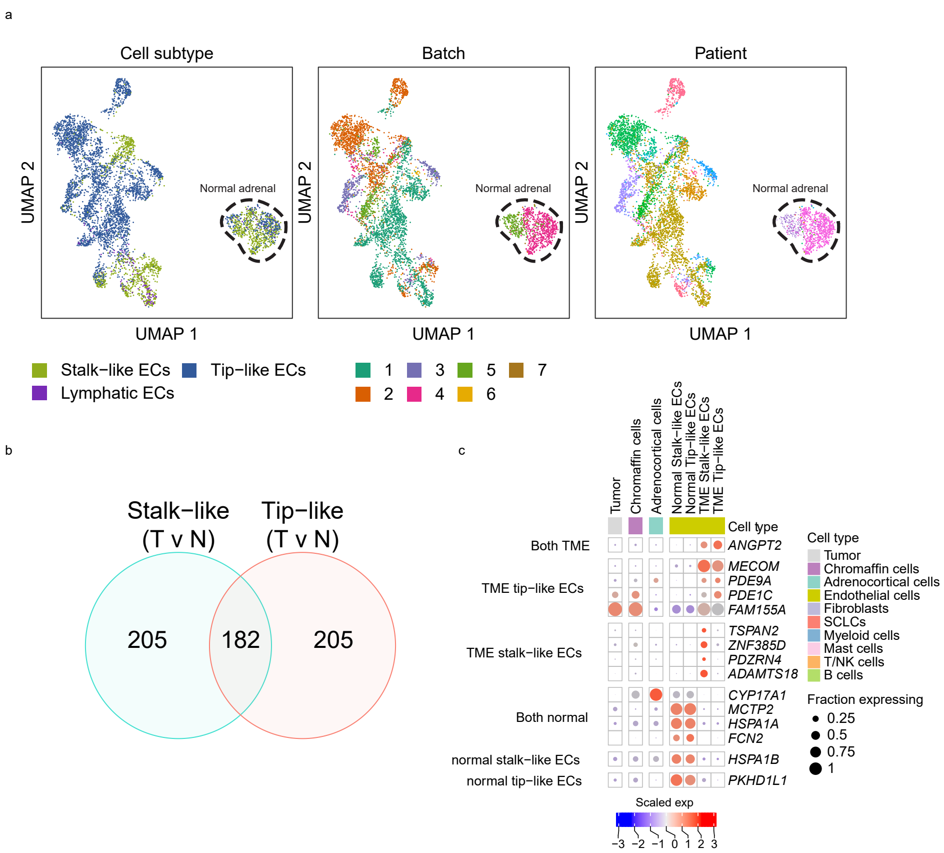

Supplementary Figure 8. Tumor-normal comparison within endothelial cell subsets. **a**, The same UMAP projection as shown in Figure 3d, colored by endothelial cell subtype, processing batch and patient. **b**, Venn-diagram showing the overlap between DEGs from comparisons between tumor-associated tip-like ECs (TME tip-like ECs) versus normal tip-like ECs, and tumor-associated stalk-like ECs (TME stalk-like ECs) versus normal stalk-like ECs (differential gene expression was decided using empirical Bayes moderated t-statistics, significant results were those with a Benjamini-Hochberg adjusted P-value < 0.05 and absolute $log_2FC > 0.5$). **c**, Dotplot showing gene expression for top DEGs from the DE analyses in (b).

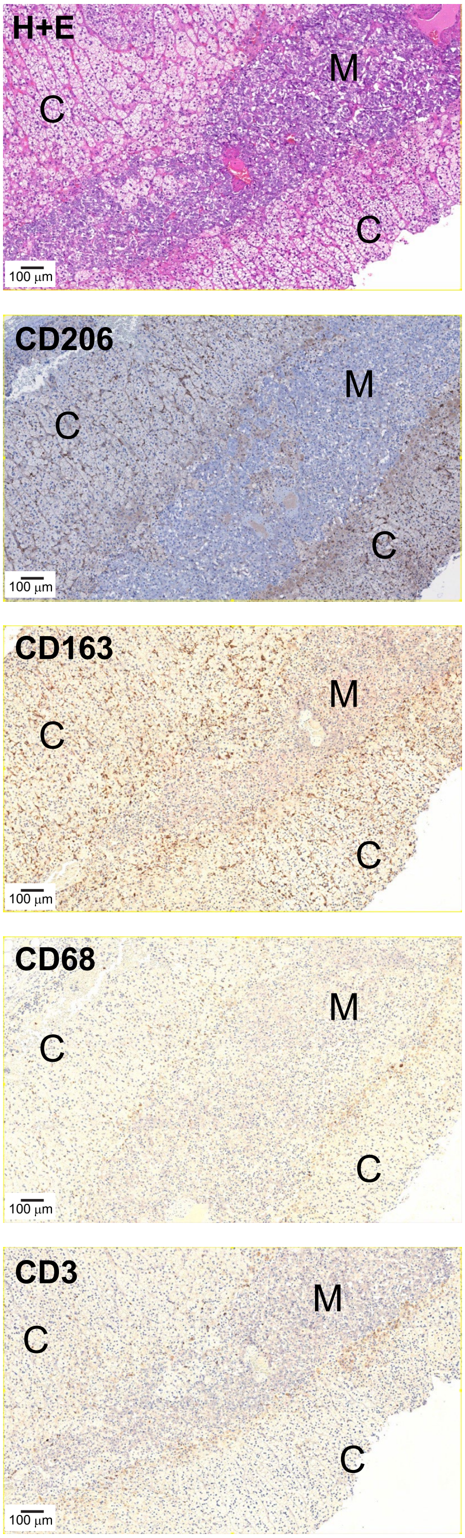

Supplementary Figure 9. Hematoxylin and eosin plus immunohistochemical staining of macrophage markers (CD206, CD168, CD68) and T cell marker (CD3) in a normal adrenal tissue (n=2, representative sections from a single sample shown). CD206+ and CD163+ cells were more numerous in the adrenal cortex compared to the medulla. Few CD68+ and CD3+ cells were observed in either the cortex or medulla regions. Scale bar indicates 100 micrometers.

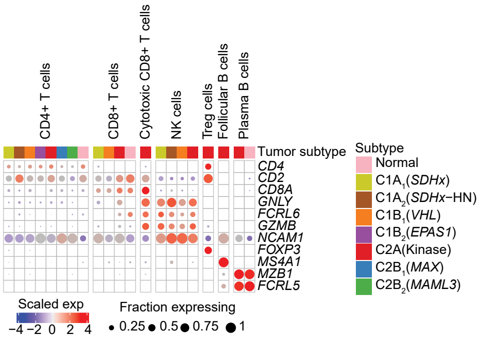

Supplementary Figure 10. Expression of lymphocyte marker genes in the immune component of PPGL. Dotplot illustrating snRNA-seq expression of lymphocyte marker genes. Dot color indicates the mean gene expression across all cells of a given cell-type within a given subtype, values are scaled and centered. Dot size indicates the proportion of cells of the indicated cell-type with detectable expression of the gene. PPGL subtypes with less than 20 cells representing an immune cell type are excluded from the plot.

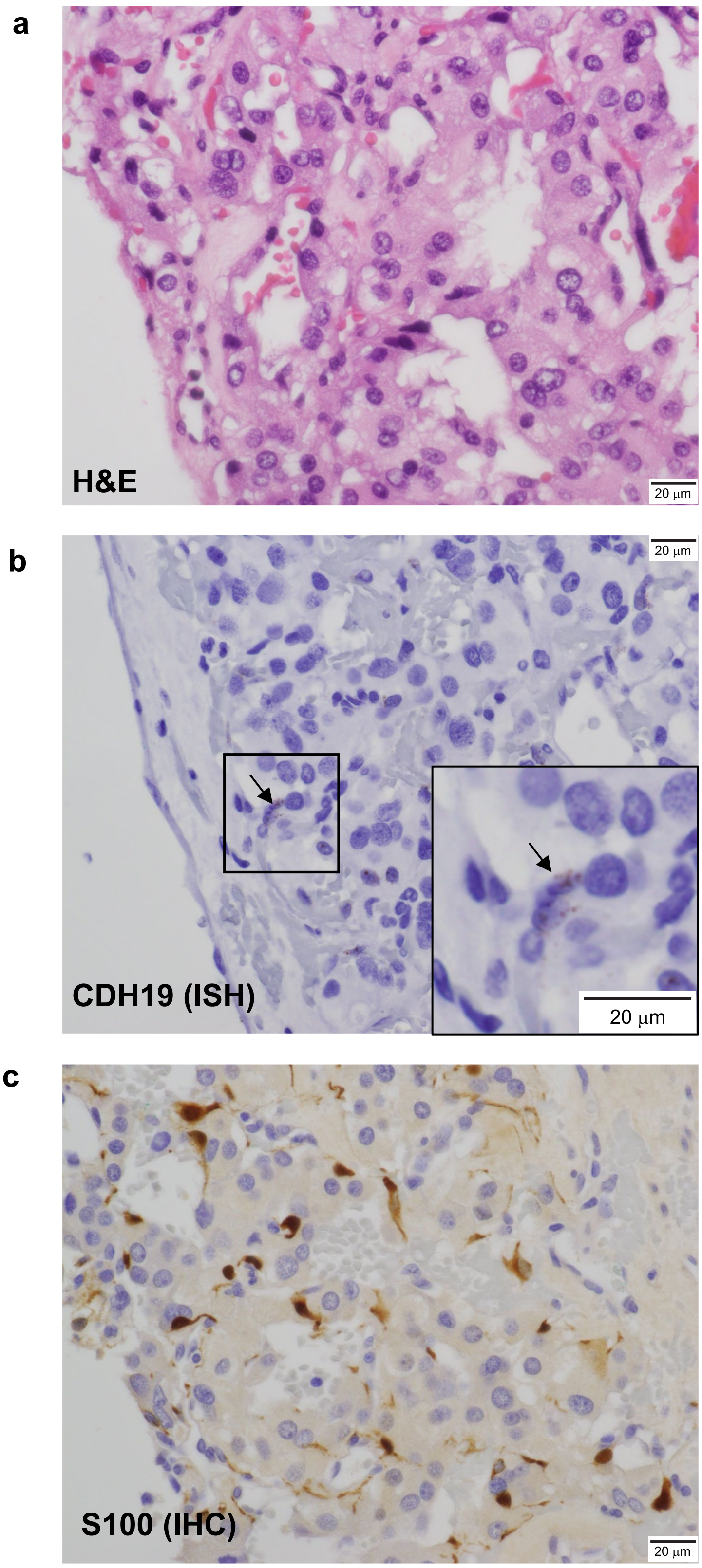

Supplementary Figure 11. CDH19 ISH (RNAscope) and S100 immunohistochemistry staining on tumor P018-PGL1. **a**, hematoxylin and eosin stained section (n=1). **b**, CDH19 ISH (n=1). **c**, S100 IHC (n=1). Scale bar indicates 20 micrometers.

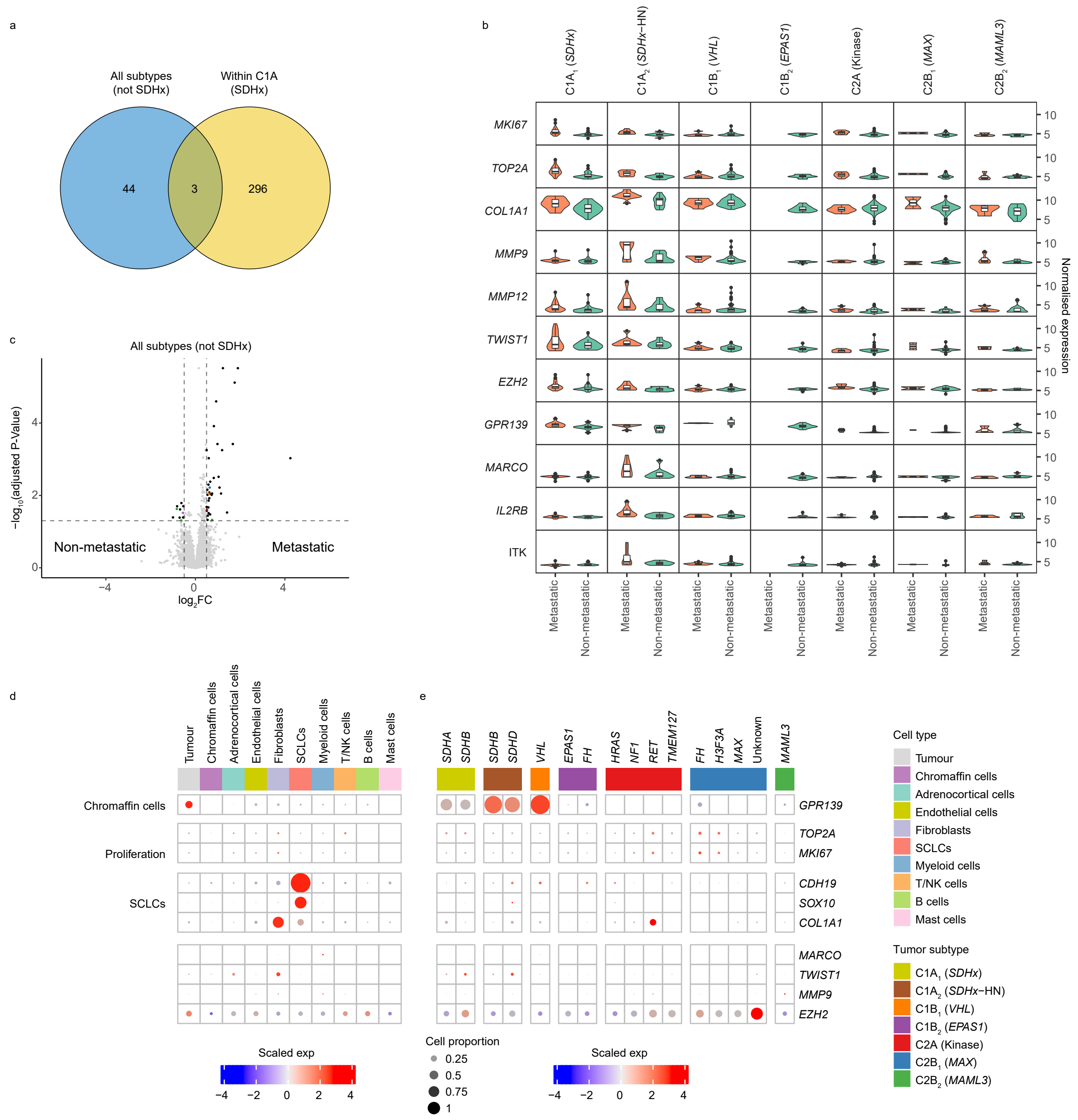

Supplementary Figure 12. Differential gene expression patterns in metastatic and non-metastatic PCPG. **a**, Venn-diagram illustrating the overlap between genes that were differentially expressed in metastatic vs non-metastatic PCPG across all subtypes other than C1A tumor, and genes that were differentially expressed in metastatic vs non-metastatic PCPG in just the C1A tumors (BH-adjusted P value < 0.05, log2FC > 0.5). **b**, Violin plots of gene expression for genes detected as differentially expressed, plotted individually for each tumor subtype (total samples: 382; samples per group: $C1A_1$-metastatic=23, $C1A_2$-metastatic=8, $C1B_1$-metastatic=8, C2A-metastatic=7, $C2B_1$-metastatic=2, $C2B_2$-metastatic=4, $C1A_1$-non-metastatic=31, $C1A_2$-non-metastatic=9, $C1B_1$-non-metastatic=69, $C1B_2$-non-metastatic=19, C2A-non-metastatic=145, $C2B_1$-non-metastatic=48, $C2B_2$-non-metastatic=9). The lower and upper hinges of each boxplot correspond to the first and third quartiles, respectively, and the median value is marked. The whiskers extend from the hinges to the largest and smallest value no greater than 1.5 times the interquartile range above or below the upper and lower hinges, respectively. Values beyond the whisker extents are deemed outliers and are plotted individually. **c**, Volcano plot indicating differentially expressed genes in the bulk RNA-seq cohort for metastatic vs non-metastatic PCPG within all subtypes other than the C1A SDHx subtypes. **d**,**e**, Dot plot describing snRNA-seq expression of genes that were detected as differentially expressed in the bulk RNA-seq comparison of metastatic vs non-metastatic PCPG in the C1A (SDHx) cluster tumors. Expression is shown in all normal cell types and aggregated tumor nuclei (d) and in all tumor nuclei aggregated by tumor genotype and subtype (e).
