# Supplementary material for: Single-nuclei and bulk-tissue gene-expression analysis of pheochromocytoma and paraganglioma links disease subtypes with tumor microenvironment
Source: Nat Commun. 2022 Oct 21;13:6262. doi: 10.1038/s41467-022-34011-3 (PMC9587261; doi:10.1038/s41467-022-34011-3)
Supplement: Supplementary file 3 — Description of Additional Supplementary Files [file 41467_2022_34011_MOESM3_ESM.pdf]

Description of Additional Supplementary Files

File Name: Supplementary Data 1
Description: Patient sample metadata for samples analyzed by snRNA-seq.

File Name: Supplementary Data 2
Description: Cell type composition of each snRNA-seq sample.

File Name: Supplementary Data 3
Description: Sample metadata for samples analyzed with bulk RNA sequencing.

File Name: Supplementary Data 4
Description: Statistical testing for genotype-subtype associations (Fisher's exact test, Benjamini-Hochberg multiple testing correction).

File Name: Supplementary Data 5
Description: "One vs rest" differential gene expression analysis for each subtype. Performed on PCPG bulk-tissue RNA-seq.

File Name: Supplementary Data 6
Description: Differential expression analysis of pseudobulk data contrasting PCPG and NAM tissues by subtype or major normal cell type

File Name: Supplementary Data 7
Description: Differential expression analysis of pseudobulk data comparing macrophages, tip-like endothelial cells and stalk-like endothelial cells in tumor tissue versus NAM tissue.

File Name: Supplementary Data 8
Description: Significant differentially abundant cell types and cell subsets in PCPG tumor subtypes

File Name: Supplementary Data 9
Description: Cell interactions determined by the Network Analysis Toolkit for the Multicellular Interactions (NATMI) algorithm

File Name: Supplementary Data 10
Description: Classification of single neoplastic cells using normal fetal reference

File Name: Supplementary Data 11
Description: Gene set modules for fetal adrenal cells (extracted from Supplementary Table 5 Janksy et al 2021)

File Name: Supplementary Data 12
Description: Differentially expressed genes in metastatic PCPG compared to non-metastatic PCPG

File Name: Supplementary Data 13
Description: Gene set modules for stromal and immune cell types
