# Supplementary material for: Single-nuclei and bulk-tissue gene-expression analysis of pheochromocytoma and paraganglioma links disease subtypes with tumor microenvironment
Source: Nat Commun. 2022 Oct 21;13:6262. doi: 10.1038/s41467-022-34011-3 (PMC9587261; doi:10.1038/s41467-022-34011-3)
Supplement: Supplementary file 17 — Reporting Summary [file 41467_2022_34011_MOESM17_ESM.pdf]

nature portfolio

Corresponding author(s): Richard W Tothill

Last updated by author(s): 9/15/2022

Reporting Summary

Nature Portfolio wishes to improve the reproducibility of the work that we publish. This form provides structure for consistency and transparency in reporting. For further information on Nature Portfolio policies, see our Editorial Policies and the Editorial Policy Checklist.

Statistics

For all statistical analyses, confirm that the following items are present in the figure legend, table legend, main text, or Methods section.

n/a	Confirmed	
☐	☒	The exact sample size (*n*) for each experimental group/condition, given as a discrete number and unit of measurement
☐	☒	A statement on whether measurements were taken from distinct samples or whether the same sample was measured repeatedly
☐	☒	The statistical test(s) used AND whether they are one- or two-sided<br>*Only common tests should be described solely by name; describe more complex techniques in the Methods section.*
☐	☒	A description of all covariates tested
☐	☒	A description of any assumptions or corrections, such as tests of normality and adjustment for multiple comparisons
☐	☒	A full description of the statistical parameters including central tendency (e.g. means) or other basic estimates (e.g. regression coefficient) AND variation (e.g. standard deviation) or associated estimates of uncertainty (e.g. confidence intervals)
☐	☒	For null hypothesis testing, the test statistic (e.g. *F*, *t*, *r*) with confidence intervals, effect sizes, degrees of freedom and *P* value noted<br>*Give P values as exact values whenever suitable.*
☒	☐	For Bayesian analysis, information on the choice of priors and Markov chain Monte Carlo settings
☐	☒	For hierarchical and complex designs, identification of the appropriate level for tests and full reporting of outcomes
☐	☒	Estimates of effect sizes (e.g. Cohen's *d*, Pearson's *r*), indicating how they were calculated

Our web collection on statistics for biologists contains articles on many of the points above.

Software and code

Policy information about availability of computer code

Data collection

Data was generated using the 10x Chromium Single Cell 5' Library & Gel Bead Kit (PN-1000002 following the manufacturer’s recommendations (10x Genomics, USA). Once processed, snRNA-Seq libraries were sequenced in multiple batches on the Illumina Nova-Seq 6000 (Illumina, USA) using 150 bp paired-end sequencing. scRNA-seq binary base calls (BCL) files were demultiplexed and converted into FASTQ files using BCLtoFastq.

Data analysis

Customs analysis scripts are available at https://github.com/UMCCR-RADIO-Lab/snRNA-seq-atlas-of-pheochromocytoma-and-paraganglioma.
Published software used included
affy (www.bioconductor.org/packages/release/bioc/html/affy.html)
AnnotationDbi (bioconductor.org/packages/release/bioc/html/AnnotationDbi.html)
Cellranger (support.10xgenomics.com/single-cell-gene-expression/software/pipelines/latest/installation)
ComplexHeatmap (www.bioconductor.org/packages/release/bioc/html/ComplexHeatmap.html)
ConsensusClusterPlus (bioconductor.org/packages/release/bioc/html/ConsensusClusterPlus.html)
EdgeR (bioconductor.org/packages/release/bioc/html/edgeR.html)
InferCNV (bioconductor.org/packages/release/bioc/html/infercnv.html)
Limma (bioconductor.org/packages/release/bioc/html/limma.html)
NATMI (github.com/asrhou/NATMI)
preprocessCore (www.bioconductor.org/packages/release/bioc/html/preprocessCore.html)
Python (www.python.org/)
R (www.r-project.org/)
scMatch (github.com/asrhou/scMatch)
Scrublet (github.com/swolock/scrublet)
SCTransform (github.com/ChristophH/sctransform)

Seurat (cloud.r-project.org/web/packages/Seurat/index.html)
Speckle (https://github.com/Oshlack/speckle)
Harmony (https://github.com/immunogenomics/harmony)
uwot (cran.r-project.org/web/packages/uwot/index.html)

For manuscripts utilizing custom algorithms or software that are central to the research but not yet described in published literature, software must be made available to editors and reviewers. We strongly encourage code deposition in a community repository (e.g. GitHub). See the Nature Portfolio guidelines for submitting code & software for further information.

Data

Policy information about availability of data

All manuscripts must include a data availability statement. This statement should provide the following information, where applicable:

- Accession codes, unique identifiers, or web links for publicly available datasets
- A description of any restrictions on data availability
- For clinical datasets or third party data, please ensure that the statement adheres to our policy

The raw snRNA-seq as well as bulk-tissue RNA-seq data generated in this study have been deposited in the European Genome-Phenome Archive (EGA) under accession code EGAS00001005861/ EGAD00001008403 (https://ega-archive.org/studies/EGAS00001005861). The data are available under restricted access as it is potentially identifiable based on patient genotype. Access can be obtained by researchers upon application through EGA to the study data access committee (DAC) of the University of Melbourne. The DAC will attempt to provide a response to all applications within ten days of submission and render a final decision within no more than four weeks. Once the DAC has in principle approved an application a data transfer agreement (DTA) will be mutually agreed and executed between institutions and data will then be made available through EGA. The remaining data are available within the article and supplementary information. Source data required for the reproduction of figures presented in this study are available from figshare (https://dx.doi.org/10.6084/m9.figshare.21080476). The publicly available microarray datasets used in this study are available from the Gene Expression Omnibus (accession numbers GSE131907 [https://www.ncbi.nlm.nih.gov/geo/query/acc.cgi?acc=GSE131907]; [30], GSE146771 [https://www.ncbi.nlm.nih.gov/geo/query/acc.cgi?acc=GSE146771]; 31], GSE2841 [https://www.ncbi.nlm.nih.gov/geo/query/acc.cgi?acc=GSE2841]; [9], GSE19422 [https://www.ncbi.nlm.nih.gov/geo/query/acc.cgi?acc=GSE19422]; [84], GSE19987 [https://www.ncbi.nlm.nih.gov/geo/query/acc.cgi?acc=GSE19987]; [85], GSE39716 [https://www.ncbi.nlm.nih.gov/geo/query/acc.cgi?acc=GSE39716]; [86], GSE50442 [https://www.ncbi.nlm.nih.gov/geo/query/acc.cgi?acc=GSE50442],; [86], GSE51081 [https://www.ncbi.nlm.nih.gov/geo/query/acc.cgi?acc=GSE51081]; [87], and GSE67066 [https://www.ncbi.nlm.nih.gov/geo/query/acc.cgi?acc=GSE67066]; [88]) and ArrayExpress (accession number E-MTAB-733 [https://www.ebi.ac.uk/arrayexpress/experiments/E-MTAB-733]; [58]). Additionally, publicly available Affymetrix Cytoscan HD array data is available from the Gene Expression Omnibus under accession numbers GSE61594 [https://www.ncbi.nlm.nih.gov/geo/query/acc.cgi?acc=GSE61594]; [77] and GSE94378 [https://www.ncbi.nlm.nih.gov/geo/query/acc.cgi?acc=GSE94378]; [26].

Field-specific reporting

Please select the one below that is the best fit for your research. If you are not sure, read the appropriate sections before making your selection.

☒ Life sciences ☐ Behavioural & social sciences ☐ Ecological, evolutionary & environmental sciences

For a reference copy of the document with all sections, see nature.com/documents/nr-reporting-summary-flat.pdf

Life sciences study design

All studies must disclose on these points even when the disclosure is negative.

Sample size	No statistical method for sample size determination was performed. Sample size was dictated by the availability of the rare cancer tissue samples and the financial resources for analysis. Samples were selected to represent a broad spectrum of tumor genotypes. Sufficient biological replicates were used for statistical analysis to identify differential gene expression sample groups.
Data exclusions	snRNA-seq analysis<br>Removal of cells if a) raw counts for each sample were annotated with ‘doublet scores’ using Scrublet (version 0.2.1) removing any cells exceeding doublet scores of 2 median absolute deviations (MAD) b) if the percent of total read counts belonging to mitochondrial genes exceeded 5 MAD values. c) Within each sample, cells were filtered out if their detected gene count or total counts were lower than a prescribed threshold with respect to cell type where a threshold of -4 MADs was chosen for cells classified as B cells, T cells, mast cells or NK cells, otherwise a threshold of -2.5 MADs was used for all other cell types.<br>Inspection of gene-expression between cell types within individual samples was done to exclude genes associated with potential ambient RNA originating from an unrelated cell types. We found genes highly expressed in adrenocortical cells were overrepresented in chromaffin cells from normal adrenal medulla (NAM) tissues therefore post-hoc filtering of these adrenocortical genes was performed for comparison of neoplastic nuclei and normal chromaffin cell nuclei. Therefore adrenocortical genes were identified and excluded if they were a) underexpressed in neoplastic cells compared to NAM chromaffin cells in 6 of 7 PCPG subtype comparisons (logFC<0, Benjamini-Hochberg adjusted p<0.05) and b) overexpressed in the adrenocortical cells versus all other cell types (logFC>0, Benjamini-Hochberg adjusted p<0.05).<br>Bulk RNA microarray gene-expression<br>Low quality Affymetrix array data from published studies involving GEO accession GSE19987 and GSE2841 were removed as they had significantly higher than average normalized unscaled standard errors (NUSE) and clustered as discrete group away from other sample data by UMAP.
Replication	No technical replication was performed for snRNA-seq, immunohistochemistry or in situ hybridization experiments.
Randomization	Samples were randomised before generation of data using snRNA-seq method.

Blinding	No blinding of samples was done as it was impractical to do this given knowledge of sample characteristics was required for allocation to sample groups by the research scientists undertaking the analysis.

Reporting for specific materials, systems and methods

We require information from authors about some types of materials, experimental systems and methods used in many studies. Here, indicate whether each material, system or method listed is relevant to your study. If you are not sure if a list item applies to your research, read the appropriate section before selecting a response.

Materials & experimental systems

n/a	Involved in the study	
☐	☒	Antibodies
☒	☐	Eukaryotic cell lines
☒	☐	Palaeontology and archaeology
☒	☐	Animals and other organisms
☐	☒	Human research participants
☒	☐	Clinical data
☒	☐	Dual use research of concern

Methods

n/a	Involved in the study	
☒	☐	ChIP-seq
☒	☐	Flow cytometry
☒	☐	MRI-based neuroimaging

Antibodies

Antibodies used	S100 (cat no: Z3011; Dako, USA), Dilution: 1:1000<br>CD3 (SP7) (cat no: Ab16669, Abcam, United Kingdom), Dilution: 1:200<br>CD68 (514H12)(cat no: MA1-80133, Thermofisher Scientific, USA), Dilution: 1:100<br>CD206 (cat no: Ab64693, Abcam, United Kingdom), Dilution: 1:10000<br>CD163 (MRQ-26) (cat no: CM163M14, Millipore Sigma, USA), Dilution: 1:200
Validation	The s100 antibody is routinely used for clinical diagnostic purposes (IHC) at Department of Anatomical Pathology, Royal North Shore Hospital, St Leonards NSW, Australia and validated on Appendix/colon/skin. CD3 (SP7) IHC validated by supplier on human tonsil. Cited in 1678 publications. CD68 IHC (514H12) validated by supplier on human tonsil. Cited by 2 publications. CD206 (cat no: Ab64693) IHC validated in Human liver and lung tissue. Cited by 427 publications. CD163 (MRQ-26) IHC validated by supplier in inflamed human tissue and human liver. Cited by 5 publications

Human research participants

Policy information about studies involving human research participants

Population characteristics	Patient cohort used for sn-RNA-seq included 18 patients with pheochromocytomas, 7 with abdominal-thoracic paraganglioma , 5 with head and neck paraganglioma and two normal adrenals. Hereditary PCPG genotypes included FH (n=2), VHL (n=2), MAX (n=2), RET (n=2), SDHA (n=1), SDHB (n=3), SDHD (n=4), TMEM127 (n=1).
Recruitment	Patients were recruited to tissue biobanks under IRB approved protocols and underwent surgery for the relevant cancer type or a corrective clinical procedure in the case of normal tissue collection. There were no other selection criteria.
Ethics oversight	Research was done under a protocol approved by the human research ethics committee at Peter MacCallum Cancer Centre and under the guidelines of the National Health and Medical Research Council in accordance with the Helsinki Declaration of 1975, as revised in 1983. All patients provided written informed consent for the use of their deidentified biospecimens for research purposes. No compensation was provided for provision of samples. Patient samples were collected under protocols approved by the respective institutional review boards (IRB). Organizations contributing patient samples included the Victorian Cancer Biobank under protocols approved at Austin Health, Melbourne Health and Monash Health (n=4), the Peter MacCallum Cancer Centre (n=4), Kolling Institute Neuroendocrine Tumor Bank under a protocol approved at North Sydney Local Health District (n=8), National Institute of Health (n=10), University of Colorado (n=1), University of Texas Health Science Center at San Antonio (n=2), Tufts Medical Centre (n=1), and Palacky University (n=2)

Note that full information on the approval of the study protocol must also be provided in the manuscript.
